# Exploring the association between triglyceride-glucose index and thyroid function

**DOI:** 10.1186/s40001-023-01501-z

**Published:** 2023-11-10

**Authors:** Hui Cheng, Yanyan Hu, Haoran Zhao, Guowei Zhou, Gaoyuan Wang, Chaoqun Ma, Yan Xu

**Affiliations:** 1 Department of General Surgery, Affiliated Hospital of Nanjing University of Chinese Medicine, Jiangsu Province Hospital of Chinese Medicine, No.155, Hanzhong Road, Qinhuai District, Nanjing, 210029 Jiangsu People’s Republic of China; 2 Nursing College, Nanjing University of Chinese Medicine, Nanjing, Jiangsu China; 3 Outpatient Department, Nanjing Hospital Affiliated to Nanjing University of Chinese Medicine, The Second Hospital of Nanjing, No.1, Zhongfu Road, Gulou District, Nanjing, 210003 Jiangsu People’s Republic of China

**Keywords:** Triglyceride-glucose index, Cross-sectional analysis, Thyroid parameter, Low-normal thyroid function, NHANES

## Abstract

**Background:**

Thyroid dysfunction is associated with abnormal glucose-insulin homeostasis, and the triglyceride-glucose (TyG) index has been recommended as a convenient surrogate of insulin resistance (IR). This study aimed to investigate the relationship between TyG and thyroid function in the US population.

**Methods:**

We analyzed data from the National Health and Nutrition Examination Survey (NHANES) conducted from 2007 to 2012 in a cross-sectional manner. Aside from conventional thyroid parameters, our study evaluated the central sensitivity to thyroid hormones (THs) using the thyroid feedback quantile-based index (TFQI), thyrotropin resistance index (TT4RI), and thyrotropin index (TSHI). To evaluate peripheral sensitivity to THs, we calculated the ratio of free triiodothyronine (FT3) to free thyroxine (FT4) and the sum activity of peripheral deiodinases (SPINA-GD). In the 1848 adults, multivariable linear regression, subgroup, and interaction analyses were employed to estimate the association between TyG and thyroid parameters. The nonlinear relationship was addressed by smooth curve fittings and generalized additive models.

**Results:**

After adjusting covariates, we demonstrated a significant negative association between TyG and FT4 (β = − 0.57, *p* < 0.001), and a positive relationship between TyG and thyroid-stimulating hormone (β = 0.34, *p* = 0.037), as well as TgAb (β = 17.06, *p* = 0.005). Subgroup analysis indicated that the association between TyG and TgAb was more pronounced in the female subjects (β = 32.39, *p* < 0.001, *p for interaction* = 0.021). We also confirmed an inverse correlation between TyG and central sensitivity to THs, as assessed by TSHI and TT4RI (βTSHI = 0.12, p < 0.001; βTT4RI = 2.54, *p* = 0.023). In terms of peripheral sensitivity to THs, we found a significant positive correlation between TyG and FT3/FT4 (β = 0.03, *p* = 0.004), and SPINA-GD (β = 2.93, *p* = 0.004).

**Conclusion:**

The present study established a noteworthy association between TyG and thyroid parameters, indicating a strong link between IR and thyroid dysfunction. Further investigations are warranted to validate these results.

**Supplementary Information:**

The online version contains supplementary material available at 10.1186/s40001-023-01501-z.

## Introduction

Insulin resistance (IR) is a condition in which tissues are not responding properly to insulin [1], and it has been identified as a major cause of numerous diseases, including type 2 diabetes (T2DM), cardiovascular diseases (CVD), polycystic ovary syndrome (PCOS), and dyslipidemia [2–4]. Currently, the hyperinsulinemic-euglycemic clamp (HEC) technique is accepted as the “gold standard” for measuring IR [5], nevertheless, this method requires a great deal of time, effort, and expense, which limits its clinical applications [6]. Therefore, researchers are dedicated to developing the surrogate indexes of IR, including homeostatic model assessment of IR (HOMA-IR) [7], metabolic score for IR (METS-IR) [8], quantitative insulin sensitivity check index (QUICKI) [5], and Matsuda index [9]. In recent studies, the triglyceride-glucose (TyG) index, determined from fasting plasma glucose (FPG) and triglycerides (TG), has been proposed as a convenient and reliable method to evaluate peripheral IR [10], as well as a potential risk marker of various diseases [11–14].

Thyroid hormones (THs) are crucial for the regulation of multiple metabolic processes [15], and it has been suggested that thyroid dysfunction can contribute to abnormal glucose homeostasis [16]. It is known that overproduction of THs can affect the alternation of insulin signaling and glucose metabolism, and clinical and experimental hyperthyroidism often coincides with impaired glucose tolerance [16]. Previous studies have also revealed the tight linkage between hypothyroidism and metabolic disorders [17, 18], and even the modest increases in thyroid-stimulating hormone (TSH) levels are correlated with the presence of IR [19]. Moreover, it has also been reported that IR is associated with low-normal thyroid function, indicated by low levels of free thyroxine (FT4) and/or high concentrations of TSH [20, 21]. However, the research investigating the relationship between THs and IR had conflicting results.

Only a few studies have investigated the relationship between TyG and thyroid parameters, which used only FT4 and TSH levels to assess thyroid function [22, 23]. Thyroid antibodies were not involved in this research, not to mention sensitivity to thyroid hormone indices, which are considered to reveal the complex correlation between THs [24]. In this study, the association between TyG and thyroid parameters was investigated in a nationally representative sample of adults from the United States (US) using the National Health and Nutrition Examination Survey (NHANES) from 2007 to 2012.

## Materials and methods

### Study population

In this study, data were obtained from the NHANES, a cross-sectional survey conducted biennially by the Centers for Disease Control and Prevention to assess US citizens’ nutrition and health conditions. Demographic information, dietary information, physical examinations, laboratory data, and multiple questionnaires are among the main sections of the database. The Institutional Review Board (IRB) of the National Center for Health Statistics (NCHS) has approved the research protocol of the NHANES before interviews or data collection begins, and the participants provided written informed consent. It is worth mentioning that this analysis exclusively relied on publicly accessible data, and no ethical approval was needed.

**Fig. 1:**
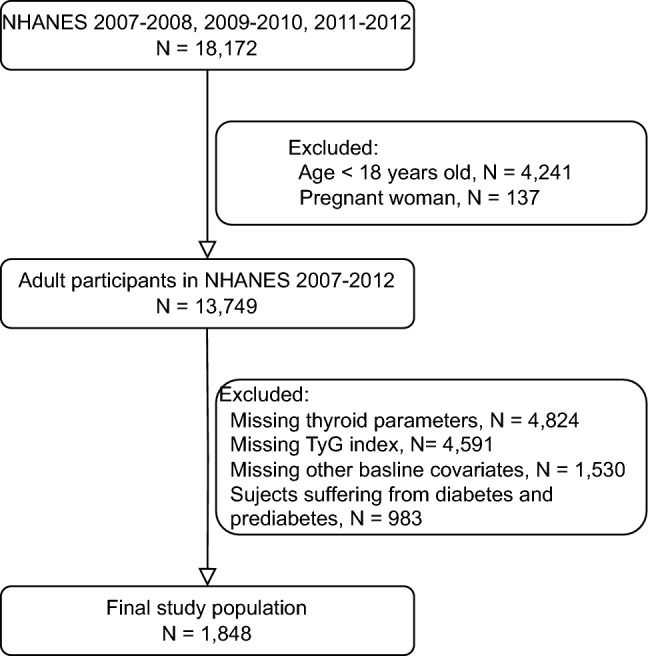
Study flowchart. NHANES, National Health and Nutrition Examination Survey; TyG, Triglyceride-glucose

Three waves of the NHANES were pooled for this study, including NHANES 2007–2008 (‘‘E’’ data), NHANES 2009–2010 (‘‘F’’ data), and NHANES 2011–2012 (‘‘G’’ data). The present study utilized NHANES operation manuals to access variables, which were available on the NHANES website (https://www.cdc.gov/nchs/nhanes/). We included participants who did not have a pregnancy, were at least 18 years old, and had laboratory data regarding TyG and thyroid function (Fig. 1). Furthermore, to avoid potential bias, the subjects suffering from diabetes or prediabetes [including impaired fasting glucose (IFG), and impaired glucose Subjects tolerance (IGT)] were excluded.

### Measurement of TyG and thyroid outcomes

TyG was calculated by the formula of Ln[TG (mg/dL) × FPG (mg/dL)/2] [25]. In the present study, thyroid function parameters were assessed including free triiodothyronine (FT3), FT4, TSH, anti-thyroglobulin antibody (TgAb), and anti-thyroperoxidase antibody (TPOAb), and the NHANES Laboratory/Medical Technologists Procedures Manual (LPM) provides detailed specimen collection and processing instructions [26]. According to the “Thyroid profile” section in the laboratory data obtained from NHANES 2007–2012, the measurement of FT3 (reference range, 2.5–3.9 pg/mL) and FT4 (reference range, 0.6–1.6 ng/dL) involved a competitive two-step enzyme immunoassay, wherein specific antibodies were introduced to the sample and subsequently combined with the chemiluminescent substrate Lumi-Phos™ 530. The resulting sample was then measured using a luminometer, with results determined through reference to a stored, multi-point calibration curve. Notably, the stripping agent was not utilized for the FT3 and FT4 tests. TSH (reference range, 0.34–5.6 µIU/mL), on the other hand, was measured using a two-site, immune-enzymatic ("sandwich") assay of the third generation, and its concentration was determined using a multi-point calibration curve, with the addition of Lumi-Phos™ 530. Furthermore, the sequential two-step immunoenzymatic "sandwich" assay was utilized to measure TPOAb and TgAb, with reference ranges of 0–9.0 IU/mL and 0–4.0 IU/mL respectively [26].

The central sensitivity to THs was evaluated using three indices, namely thyrotropin index (TSHI), thyrotropin thyroxine resistance index (TT4RI), and thyroid feedback quantile-based index (TFQI), which were calculated according to previous studies [27], [28]:

$$ {\text{TSHI}} = {\text{ln TSH }}\left( {{\text{mIU}}/{\text{L}}} \right)\, + \,0.{1345 }*{\text{ FT4 }}\left( {{\text{pmol}}/{\text{L}}} \right) $$

$$ {\text{TT4RI}}\, = \,{\text{FT4 }}\left( {{\text{pmol}}/{\text{L}}} \right)\, \times \,{\text{TSH }}\left( {{\text{mIU}}/{\text{L}}} \right) $$

Based on FT4 and TSH quantiles transformed by empirical cumulative distribution functions (cdf), TFQI was calculated as follows [29]:

$$ {\text{TFQI}}\, = \,{\text{cdf FT4 }}\left( {{\text{pmol}}/{\text{L}}} \right) - \left[ {{1} - {\text{cdf TSH }}\left( {{\text{mIU}}/{\text{L}}} \right)} \right] $$

A higher value of TSHI or TT4RI indicates a lower central sensitivity to THs. As for TFQI, a negative value indicates greater pituitary sensitivity to THs, a positive value indicates less sensitivity and a value of 0 indicates normal sensitivity.

As for peripheral sensitivity to THs, the FT3/FT4 ratio and the sum activity of peripheral deiodinases (SPINA-GD) were applied according to the equation:

FT3/FT4 ratio = FT3 (pmol/L)/FT4 (pmol/L) [30]. An increased FT3/FT4 ratio indicates a higher activity of peripheral THs.

SPINA-GD = β_31_ (K_M1_ + [FT4]) (1 + K_30_[TBG]) [FT3]/α_31_[FT4] [31]. β_31:_ Clearance exponent for T3; K_M1_: Dissociation constant of type1 1 deiodinase; K_30_: Dissociation constant of T3 at thyroxine-binding globulin; TBG: Standard concentration of thyroxine-binding globulin; α_31:_ Dilution factor for triiodothyroine.

The secretory capacity of the thyroid gland (SPINA-GT) = β_T_ (D_T_ + [TSH]) (1 + K_41_[TBG] + K_42_[TBPA]) [FT4]/α_T_[TSH] [31]. β_T:_ Clearance exponent for T4; DT: EC_50_ for TSH; K_41_: Dissociation constant of T4 at thyroxine-binding globulin; TBG: Standard concentration of thyroxine-binding globulin; TBPA: Standard transthyretin concentration; K_42_: Dissociation constant of T4 at transthyretin; α_T_: Dilution factor for thyroxine.

Thyroid autoimmunity was defined as TPOAb titers exceeding 9.0 IU/mL and/or TgAb titers exceeding 4.0 IU/mL [32].

### Collect of demographics and social characteristics

The information regarding age, gender, race/ethnicity, education, marital status, poverty-to-income ratio (PIR), medical history (including diabetes, hypertension, and congestive heart failure), tobacco consumption, and alcohol use were collected using a standardized questionnaire. Education levels were classified into three groups: less than high school diploma, high school diploma, and more than high school diploma, and the socioeconomic status of the participants was evaluated using PIR, categorized as 0–1 and > 1. Tobacco use was classified into three categories proposed by NHANES: never, former, and current status. Participants who were never smokers reported smoking less than 100 cigarettes in their lives, whereas former smokers were defined as having smoked more than 100 cigarettes in life but not currently. Similarly, alcohol consumption was coded into never, former, and current conditions, with current alcohol users further divided into three categories: (1) heavy alcohol users (≥ 3 drinks per day or binge drinking [≥ 4 drinks on the same occasion] ≥ 5 days per month for females, ≥ 4 drinks per day or binge drinking [≥ 5 drinks on the same occasion] ≥ 5 days per month for males; (2) moderate alcohol users (≥ 2 drinks per day for females, ≥ 3 drinks per day for males, or binge drinking ≥ 2 days per month); and (3) mild alcohol users were the ones who drunk currently but did not meet heavy and moderate alcohol standards [33].

Body mass index (BMI), waist circumference (WC), and blood pressure (BP) were measured by certified technicians in Mobile Examination Centers (MECs). BMI was calculated using the following formula: BMI = body weight (kg)/height^2^ (m^2^). WC was assessed using an inelastic ruler with a minimum scale of one millimeter. Measurements were taken after a regular exhalation, while the participant stood in a natural position with legs positioned approximately 25–30 cm apart. The ruler was positioned at the midpoint of the connecting line between the upper edge of the iliac crest and the lower edge of the 12th rib, which typically represents the narrowest point of the waist. A horizontal circumference measurement was then obtained by encircling the abdomen. The final measurement was rounded to the nearest 0.1 cm [34]. Furthermore, a gender-specific cut-off of waist circumference (102 cm for males, 88 cm for females) was then involved in the subgroup analysis [35]. Following a minimum of 5 min of rest at the MEC, BP was assessed in a seated posture using a standardized mercury sphygmomanometer [36]. Furthermore, physical activity (PA) was calculated based on five categories: vigorous work activity/vigorous recreational activity, moderate work activity/moderate recreational activity, and walking/bicycle. The weekly metabolic equivalent (MET) was calculated using the NHANES recommended MET scores of 8 points for vigorous work activity/vigorous recreational activities and 4 points for moderate work activity/moderate recreational activities and walking/bicycle. Additionally, the weekly MET minutes were documented as low, intermediate, and high levels [37, 38].

### Measurement of laboratory indices

Subjects who fasted for a minimum of 8 h but less than 24 h had their levels of FPG, fasting blood insulin (FBI), total cholesterol (TC), TG, low-density lipoprotein cholesterol (LDL-C), and high-density lipoprotein cholesterol (HDL-C) estimated, which were recorded by well-trained technicians utilizing standardized laboratory methods. FPG was measured using the hexokinase assay, and FBI was determined through a two-site enzyme immunoassay. Notably, FPG and FBI were measured in the morning examination session only. HDL-C, LDL-C, TC, and TG were determined in serum using a nephelometric immunoassay on the Roche Modular P chemistry analyzer.

According to “Standard Biochemistry Profile” in laboratory data from NHANES 2007–2012, the LX20 employs an enzymatic rate technique for the quantification of alanine aminotransferase (ALT) and aspartate aminotransferase (AST) activity in serum or plasma. The concentration of blood urea nitrogen (BUN) was determined utilizing the enzymatic conductivity rate method. Creatinine was measured using a Jaffe rate method (kinetic alkaline picrate). The quantification of uric acid was conducted through a timed endpoint methodology.

Hemoglobin was measured using a single-beam photometer, while glycohemoglobin (HbA1c) measurements were performed on the A1c G7 HPLC Glycohemoglobin Analyzer. Moreover, urine iodine concentration (UIC) was measured by ICP-DRC-MS (Inductively Coupled Plasma Dynamic Reaction Cell Mass Spectroscopy) to determine iodine conditions in participants, which was classified as < 99 g/L, 99–199 g/L, and > 199 g/L in this analysis [39]. All laboratory measurements complied with the standardization and certification program. Further comprehensive details regarding the analyzers and methodologies utilized can be obtained from the laboratory method file accessible on the NHANES website [https://www.cdc.gov/nchs/nhanes/ (accessed date: 11 September 2023)].

### Definition of metabolic syndrome

Metabolic Syndrome (MetS) was defined following the updated criteria established by the National Cholesterol Education Program/Adult Treatment Panel III (NCEP-ATP III) for the American population. Specifically, individuals were classified as having MetS if they met three or more of the following components: WC ≥ 102 cm for males or ≥ 88 cm for females; BP ≥ 130/85 mmHg or receiving treatment with anti-hypertensive medications; FPG ≥ 5.6 mmol/L or receiving medications for diabetes management; TG levels ≥ 150 mg/dL or receiving medications for lipid abnormalities; and HDL levels < 40 mg/dL for males or < 50 mg/dL for females, or receiving medications for lipid abnormalities [40].

### Statistical analysis

In this analysis, continuous baseline variables were summarized using the mean ± standard deviation (SD) and compared using a one-way analysis of variance (ANOVA) analysis. Categorical variables were summarized by percentage and tested using Chi-squared statistics. The association between TyG and thyroid function was investigated using multivariable linear and logistic regression analyses. Then we performed interaction and subgroup analyses according to gender and WC category. The initial analysis (model 1) was conducted without any adjustments, while subsequent analyses involved iterative adjustments for covariates that were deemed clinically significant. Model 2 adjusted for age, gender, and race/ethnicity. Model 3 added education, PIR, alcohol use, smoke, WC, systolic blood pressure, diastolic blood pressure, physical activity level, hypertension, CVD, MetS, FBI, HbA1c, AST, ALT, BUN, HDL-C, LDL-C, TC, creatinine, uric acid, hemoglobin, and urine iodine. Moreover, the current study used smooth curve fittings and generalized additive models (GAM) to address the nonlinear relationship, and a recursive algorithm was further applied to assess inflection points. All data was analyzed using the statistical software packages R 4.3.0 (http://www.R-project.org) and EmpowerStats (http://www.empowerstats.com, X&Y Solutions, Inc., Boston, MA), with a two-tailed *p-*value < 0.05 considered statistically significant.

## Result

### Baseline characteristics of participants

The clinical and laboratory characteristics of the subjects are shown in Table 1. Based on NHANES 2007–2012, this study included 1848 participants (range: 18–80 years), of whom 966 (52.57%) were males and 882 (47.73%) were females, with a mean age of 41.44 ± 16.81 years. The mean level of TyG was 8.43 ± 0.55.

**Table 1:** Baseline characteristics of the NHANES (2007–2012) study population in TyG tertiles

Characteristics	Overall	Reference range	
		Male	Female
TyG	8.43 ± 0.55		
Demographics			
Age, years	41.44 ± 16.81		
Gender (%)			
Male	966 (52.57%)^a^		
Female	882 (47.73%)		
Race (%)			
Mexican American	292 (15.80%)		
Non-Hispanic Black	354 (19.16%)		
Non-Hispanic White	870 (47.08%)		
Other Hispanic	191 (10.34%)		
Other Race	141 (7.63%)		
Marital status (%)			
Married	891 (48.21%)		
Unmarried	813 (43.99%)		
Unknown	144 (7.79%)		
PIR (%)			
0–1	351 (18.99%)		
> 1	1360 (73.59%)		
Unknown	137 (7.41%)		
Education (%)			
< High school diploma	416 (22.51%)		
High school diploma	410 (22.19%)		
> High school diploma	1022 (55.30%)		
Alcohol use (%)			
Never	162 (8.77%)		
Former	218 (11.80%)		
Mild	548 (29.65%)		
Moderate	277 (14.99%)		
Heavy	416 (22.51%)		
Unknown	227 (12.28%)		
Smoke (%)			
Never	965 (52.22%)		
Former	347 (18.78%)		
Current	391 (21.16%)		
Unknown	145 (7.85%)		
BMI, kg/m^2^	27.07 ± 5.62		
WC, cm	93.38 ± 14.12		
SBP, mmHg	117.14 ± 15.49		
DBP, mmHg	68.44 ± 11.41		
MetS (%)			
No	1157 (84.5%)		
Yes	291 (15.75%)		
PA level (%)			
Low	614 (33.23%)		
Intermediate	618 (33.44%)		
High	616 (33.33%)		
Hypertension (%)			
No	1396 (75.54%)		
Yes	452 (24.46%)		
CVD (%)			
No	1620 (87.66%)		
Yes	84 (4.55%)		
Unknown	144 (7.79%)		
Laboratory indices			
FPG, mmol/L	5.30 ± 0.43	3.33–5.56	3.33–5.56
FBI, pmol/L	64.10 ± 46.98	12–150	12–150
HbA1c, %	5.34 ± 0.36	4.3–6.0	4.3–6.0
ALT, u/L	25.53 ± 27.39	11–47	7–30
AST, u/L	26.03 ± 27.27	13–33	13–33
Creatinine, umol/L	75.01 ± 18.06	61.88–114.92	53.04–97.24
Uric acid, umol/L	318.17 ± 78.32	214.13–499.63	172.49–446.10
BUN, mmol/L	4.28 ± 1.53	2.14–8.22	2.14–8.22
TC, mg/dL	192.95 ± 40.06	< 200	< 200
HDL, mg/dL	55.25 ± 15.16	> 40	> 50
LDL, mg/dL	114.66 ± 34.55	< 135	< 135
Hemoglobin, g/dL	14.37 ± 1.51	19–65 years: 13.1–17.5 > 65 years: 11.4–17.1	19–65 years: 10.6–15.6 > 65 years: 10.9–15.9
Urine iodine (%)			
< 99 µg/L	705 (38.15%)		
99–199 µg/L	623 (33.71%)		
> 199 µg/L	520 (28.14%)		
Thyroid function			
FT3, pg/mL	3.29 ± 0.62	2.5–3.9	2.5–3.9
FT4, pmol/L	10.36 ± 2.15	7.74–20.64	7.74–20.64
TSH, uiu/mL	1.20 ± 3.02	0.34–5.60	0.34–5.60
TFQI	0.02 ± 0.33		
TSHI	1.84 ± 0.66		
TT4RI	19.96 ± 20.88		
FT3/FT4	0.51 ± 0.21		
SPINA-GT, pmol/s	3.74 ± 50.26		
SPINA-GD, nmol/s	46.72 ± 18.94		
TgAb, iu/mL	11.47 ± 111.79	0–4.0	0–4.0
TPOAb, iu/mL	18.85 ± 87.51	0–9.0	0–9.0
Thyroid autoimmunity (%)			
No	1623 (87.82%)		
Yes	225 (12.18%)		

NHANES, National Health and Nutrition Examination Survey; TyG, Triglyceride-glucose; PIR, poverty-to-income ratio; BMI, body mass index; WC, waist circumference; SBP, systolic blood pressure; DBP, diastolic blood pressure; CVD, cardiovascular diseases; MetS, metabolic syndrome; PA, physical activity; FBI, fasting blood insulin; HbA1c, glycohemoglobin; AST, aspartate aminotransferase; ALT, alanine aminotransferase; BUN, blood urea nitrogen; HDL, high density lipoprotein; LDL, low density lipoprotein; TC, total cholesterol; FT3, free triiodothyronine; FT4, free thyroxine; TSH, thyroid-stimulating hormone; TgAb, anti-thyroglobulin antibody; TPOAb, anti-thyroperoxidase antibody; TSHI, thyrotropin index; TT4RI, thyrotropin thyroxine resistance index; TFQI, thyroid feedback quantile-based index

### Association between TyG index and thyroid function

**Fig. 2:**
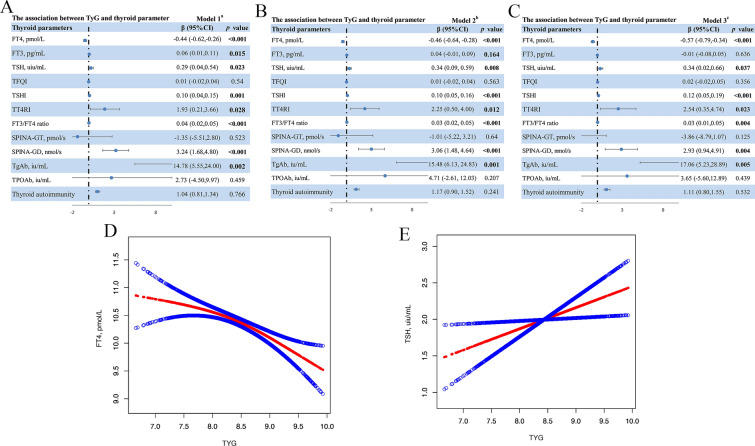
The association between TyG index and thyroid parameters. **A **Model 1: no covariates were adjusted. **B** Model 2: age, gender, and race/ethnicity were adjusted. **C** Model 3: age, gender, race/ethnicity, education, poverty-to-income ratio, alcohol use, smoke, waist circumference, systolic blood pressure, diastolic blood pressure, physical activity level, hypertension, cardiovascular disease, metabolic syndrome, fasting blood insulin, glycohemoglobin, aspartate aminotransferase, alanine aminotransferase, blood urea nitrogen, high density lipoprotein, low density lipoprotein, total cholesterol, creatinine, uric acid, hemoglobin, and urine iodin were adjusted. The solid red line represents the smooth curve fit between TyG index and FT4 (**D**), as well as TSH (**E**). Blue bands represent the 95% confidence interval from the fit. TyG, Triglyceride-glucose; FT3, free triiodothyronine; FT4, free thyroxine; TSH, thyroid-stimulating hormone; TgAb, anti-thyroglobulin antibody; TPOAb, anti-thyroperoxidase antibody; TSHI, thyrotropin index; TT4RI, thyrotropin thyroxine resistance index; TFQI, thyroid feedback quantile-based index

**Fig. 3:**
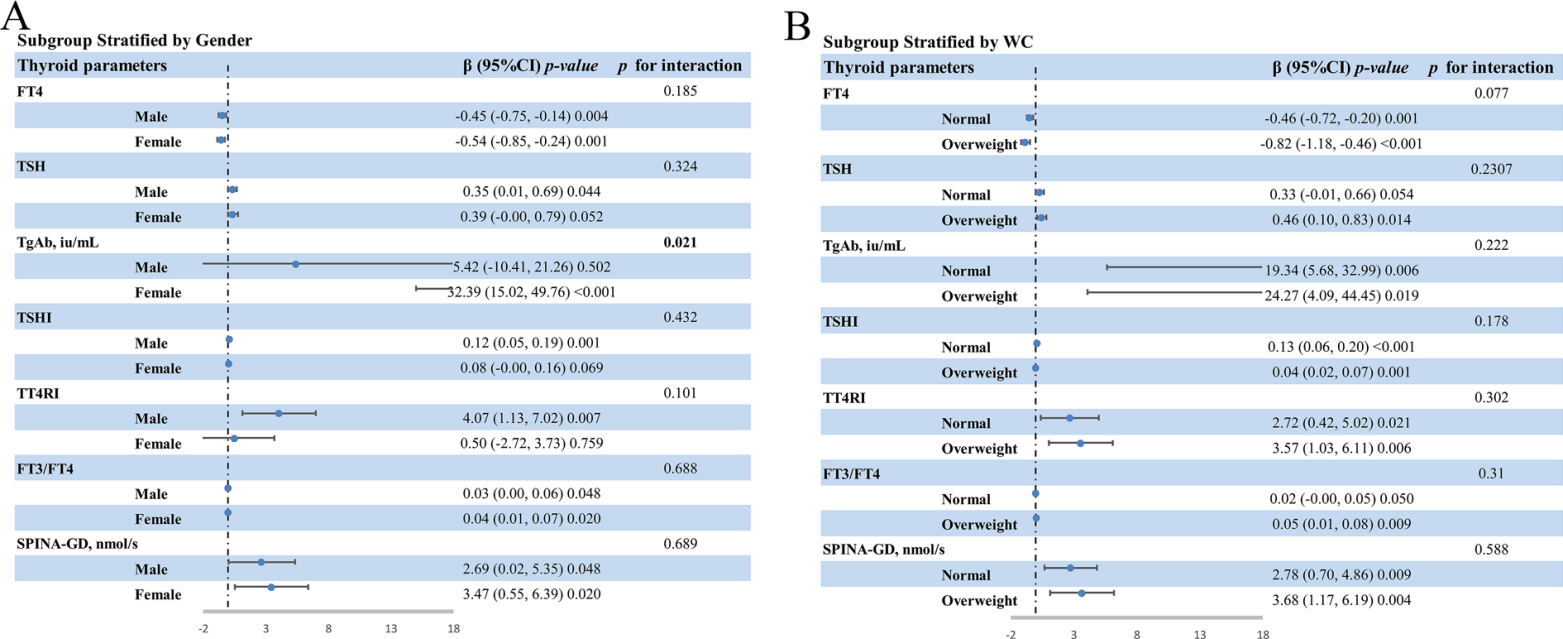
Subgroup analysis stratified by gender and WC. In the subgroup analysis of gender (**A**), age, race/ethnicity, education, poverty-to-income ratio, alcohol use, smoke, waist circumference, systolic blood pressure, diastolic blood pressure, physical activity level, hypertension, cardiovascular disease, metabolic syndrome, fasting blood insulin, glycohemoglobin, aspartate aminotransferase, alanine aminotransferase, blood urea nitrogen, high density lipoprotein, low density lipoprotein, total cholesterol, creatinine, uric acid, hemoglobin, and urine iodin were adjusted. In the subgroup analysis of WC (**B**), age, gender, race/ethnicity, education, poverty-to-income ratio, alcohol use, smoke, systolic blood pressure, diastolic blood pressure, physical activity level, hypertension, cardiovascular disease, metabolic syndrome, fasting blood insulin, glycohemoglobin, aspartate aminotransferase, alanine aminotransferase, blood urea nitrogen, high density lipoprotein, low density lipoprotein, total cholesterol, creatinine, uric acid, hemoglobin, and urine iodin were adjusted. TyG, Triglyceride-glucose; WC, waist circumference; FT4, free thyroxine; TSH, thyroid-stimulating hormone; TgAb, anti-thyroglobulin antibody; TSHI, thyrotropin index; TT4RI, thyrotropin thyroxine resistance index

As demonstrated in Fig. 2, the TyG index was significantly negatively and positively associated with FT4 and TSH, respectively. The negative association between TyG index and FT4 was present in the unadjusted model [β = − 0.44, 95% confidence interval (CI): − 0.62 to − 0.26, *p* < 0.001], model 2 (β = − 0.46, 95% CI: − 0.64 to − 0.28, *p* < 0.001), and model 3 (β = − 0.57, 95% CI: − 0.79 to − 0.34, *p* < 0.001). Similarly, the result of weighted multivariable linear regression analysis indicated a significant positive relationship between TyG index and TSH in the unadjusted model (β = 0.29, 95% CI: 0.04 to 0.54, *p* = 0.023), model 2 (β = 0.34, 95% CI: 0.09 to 0.59, *p* = 0.008), and model 3 (β = 0.34, 95% CI: 0.02 to 0.66, *p* = 0.037). Furthermore, Fig. 2 also indicated that TyG index was also significantly associated with TgAb in the unadjusted model (β = 14.78, 95% CI: 5.55 to 24.00, *p* = 0.002), model 2 (β = 15.48, 95% CI: 6.13 to 24.83, *p* = 0.001), and model 3 (β = 17.06, 95% CI: 5.23 to 28.89, *p* = 0.005). In the subgroup analysis (Fig. 3), gender and WC didn’t play an interactive role in the relationship between TyG and FT4, and TSH, while the significant association between TyG index and TgAb was more pronounced in the female subjects (β = 32.39, 95% CI: 15.02 to 49.76, *p* < 0.001, *p for interaction* = 0.021). Whereas, there was no significant association between TyG index and the levels of FT3 and TPOAb, as indicated in Fig. 2.

### Association between TyG index and sensitivity to thyroid hormone indices

A significant negative correlation was observed between TyG index and central sensitivity to THs, as assessed by TSHI and TT4RI. As shown on Fig. 2 and Additional file 1: Figure S1, TyG index showed a positive association with TSHI and TT4RI, and the significant association remained in the unadjusted model (β_TSHI_ = 0.10, 95% CI: 0.04 to 0.15, *p* = 0.001; β_TT4RI_ = 1.93, 95% CI: 0.21 to 3.66, *p* = 0.028), model 2 (β_TSHI_ = 0.10, 95% CI: 0.05 to 0.16, *p* < 0.001; β_TT4RI_ = 2.25, 95% CI: 0.50 to 4.00, *p* = 0.012), and model 3 (β_TSHI_ = 0.12, 95% CI: 0.05 to 0.19, *p* < 0.001; β_TT4RI_ = 2.54, 95% CI: 0.35 to 4.74, *p* = 0.023). However, after adjusting for confounders, there was no significant relationship between TyG index and TFQI. In terms of peripheral sensitivity to thyroid hormones, as demonstrated in Fig. 2 and Additional file 1: Figure S1, the present study found a significant positive correlation between TyG index and FT3/FT4 ratio in the unadjusted model (β = 0.03, 95% CI: 0.02 to 0.05, *p* < 0.001), model 2 (β = 0.03, 95% CI: 0.02 to 0.05, *p* < 0.001), and model 3 (β = 0.03, 95% CI: 0.01 to 0.05, *p* = 0.004). Moreover, TyG index and SPINA-GD also showed a significant relationship: unadjusted model (β = 3.24, 95% CI: 1.68 to 4.80, *p* < 0.001), model 2 (β = 3.06, 95% CI: 1.48 to 4.64, *p* < 0.001), model 3 (β = 2.93, 95% CI: 0.94 to 4.91, *p* = 0.004).

## Discussion

In the present study, a nationally representative sample of the American population was investigated to determine the relationship between TyG index and thyroid function. In the 1848 adult participants, we noted a significant association between TyG index and FT4, TSH, and TgAb, as well as the sensitivity to thyroid hormone indices throughout multiple analyses. Subgroup analysis indicated that a positive association between TyG index and TgAb existed in the female participants.

It is well known that THs and insulin signaling are interconnected, with abnormalities in one that can lead to dysregulation of the other [41]. In line with our results, a cohort study conducted by Amouzegar et al. of 2758 euthyroid participants from the Tehran Thyroid Study (TTS) to investigate the relationship between IR, as assessed by HOMA-IR, and thyroid function and indicated that low FT4 levels were independently associated with IR [42]. Likewise, an investigation of 6241 euthyroid subjects found that IR was significantly related to low normal FT4 concentrations after adjusting for age, gender, metabolic, and lifestyle factors [43]. Our work was also in agreement with those of Choi et al., who observed an inverse relationship between TyG index and FT4 after adjusting for confounders based on the Korean National Health and Nutritional Examination Survey (KNHANES) 2015 [23]. Even though T4 was regarded as a prohormone for biologically active T3, the present study showed a close relationship between TyG index and FT4, rather than FT3 [44]. It seemed logical when considering that FT4 might be a more reliable indicator of tissue thyroid status for its core mediative effect in peripheral thyroid activity and local thyroid regulatory mechanisms and that FT4 has a more efficient non-genomic capacity to drive metabolism for it is independent of nuclear receptor binding [16, 20, 45].

Research regarding the relationship between IR and TSH levels has produced conflicting results. In a cross-sectional study of a Netherlands population, Roos et al. found that the HOMA-IR index and TSH concentrations were not significantly related after further adjustment for confounders [20]. Similarly, Ruhla et al. also observed a weak relationship between TSH levels and IR, which was no longer significant after excluding the individuals suffering impaired glucose tolerance (IGT) [46]. The results of the investigation by Amouzegar et al. indicated that, despite a significant positive association observed in linear regression analysis for serum TSH levels and IR, it was not significant in the logistic regression model [42]. Inversely, in a retrospective study performed by Zhu et al., TSH was independently associated with IR, and a significant correlation was prominent in the participants with diabetes and high HbA1c levels [47]. Likewise, Ambrosi et al. also observed higher TSH levels in the obese subjects with IR, and that TSH was positively and negatively associated with HOMA-IR index and QUICKI index, respectively [48]. After adjusting for confounding factors, the current investigation revealed a significant positive relationship between TyG index and TSH, which was consistent with previous studies conducted on Korean cohorts [22, 23]. Such discrepancies may have been due to the application of different inclusion criteria, study designs, ethnicity, and assay of laboratory indices. Furthermore, these divergences may also be attributed to the application of different surrogate IR indexes. Unlike the more popular HOMA-IR index, which is primarily used to estimate hepatic IR, TyG index is considered the reliable indicator of peripheral IR [10, 49].

In general, our results, along with other studies suggested a close link between IR and low normal thyroid function, overt or subclinical hypothyroidism [20, 21, 43, 46, 50, 51]. In addition, this investigation also showed a threshold effect and a saturation effect of the association between FT4, TSH, and TyG index, respectively, suggesting that hyper- and hypothyroidism might both be associated with IR, but through different mechanisms. Moreover, the central and peripheral mechanisms should be taken into account in light of the significant associations observed between the TyG index and TSHI, TT4RI, FT3/FT4 ratio, and SPNIA-GD. In instances of hypo- and hyperthyroidism, THs possess the capacity to regulate insulin secretion both directly and indirectly, through the suppression of glucose-induced insulin secretion and the reduction of β-cell responsiveness [52, 53]. There is evidence that hypothyroidism can reduce glucose absorption from the gastrointestinal tract, increase insulin levels, and suppress hepatic glucose production [16, 54]. Whereas, as a result of hyperthyroidism, glucose depletion increases substantially, increasing insulin demand in peripheral tissues, which is generated by insulin-stimulated glucose oxidation, and ultimately leads to a disruption in hepatic insulin sensitivity [16, 55]. Furthermore, in a hyperthyroid state, hepatic gluconeogenesis is augmented through canonical transcription-mediated THs signaling, which activates gluconeogenic-drive proteins and glucose transporters, or through secondary effects on hepatocytes, resulting in reduced hepatic insulin sensitivity [56, 57].

The findings presented in this research can be elucidated through various mechanisms. It has been reported that THs play an essential role in regulating the expression and activation of proteins related to IR, such as β_2_ adrenergic receptor (β_2_AR) and peroxisome proliferator-activated receptor (PPAR-γ) [58]. In addition, THs also increase glucose transport and glycolysis by upregulating glucose transporter type-4 (GLUT-4) and phosphoglycerate kinase genes, which further work synergistically with insulin to promote glucose disposal and utilization in peripheral tissues [59–63]. Moreover, it is plausible that the mechanism may involve a synergistic interaction between THs and other endocrine factors, such as catecholamine, adiponectin, and ghrelin, whose imbalances could potentially result in enhanced insulin sensitivity [64–67].

The findings of this study should be considered in the context of certain limitations. First, the causal relationship between thyroid parameters and IR could not be established for the cross-sectional design of NHANES, thus, a cohort study with a larger sample size and longer observation periods, along with a Mendelian randomization investigation is warranted. Furthermore, the data about medical history, PIR, education, marital status, tobacco consumption, and alcohol use were acquired through self-reported questionnaires, thereby rendering the possibility of recall bias and other potential inaccuracies. Ultimately, the TyG index is considered a noteworthy parameter with extensive biological implications that can be impacted by various endocrine factors. Unfortunately, these endocrine confounders, such as catecholamine, adiponectin, and ghrelin, were not included in NHANES.

## Conclusion

In conclusion, the current investigation, which employed a nationally representative sample of the adult population in the US, has established a strong association between the TyG index and thyroid parameters, including FT4, TSH, TgAb, TT4RI, TSHI, FT3/FT4 ratio and SPINA-GD. These findings indicate a noteworthy correlation between IR and thyroid dysfunction, necessitating additional causal investigations to validate this inference.

### Supplementary Information

**Additional file 1.** The association between TyG index and thyroid parameters.

## Data Availability

The original contributions presented in the study are included in the article/Supplementary Material. Further inquiries can be directed to the corresponding authors.
