# Correction: Exploring the association between triglyceride-glucose index and thyroid function

**DOI:** 10.1186/s40001-024-01658-1

**Published:** 2024-01-24

**Authors:** Hui Cheng, Yanyan Hu, Haoran Zhao, Guowei Zhou, Gaoyuan Wang, Chaoqun Ma, Yan Xu

**Affiliations:** 1 Department of General Surgery, Affiliated Hospital of Nanjing University, of Chinese Medicine, Jiangsu Province Hospital of Chinese Medicine, No.155, Hanzhong Road, Qinhuai District, Nanjing, 210029 Jiangsu People’s Republic of China; 2 Nursing College, Nanjing University of Chinese Medicine, Nanjing, Jiangsu China; 3 Outpatient Department, Nanjing Hospital Affiliated to Nanjing, University of Chinese Medicine, The Second Hospital of Nanjing, No.1, Zhongfu Road, Gulou District, Nanjing, 210003 Jiangsu People’s Republic of China

**Correction: European Journal of Medical Research (2023) 28:508** 10.1186/s40001-023-01501-z

In this article the affiliation details of the co-author, Yanyan-Hu was incorrectly given as ‘1, 2’ but should have been ‘2’. The author has only one affiliation as “Nursing College, Nanjing University of Chinese Medicine, Nanjing, Jiangsu, China”. The original article has been corrected.
