# Supplementary material for: Exploring the association between triglyceride-glucose index and thyroid function
Source: Eur J Med Res. 2023 Nov 10;28:508. doi: 10.1186/s40001-023-01501-z (PMC10636949; doi:10.1186/s40001-023-01501-z)
Supplement: Supplementary file 1 — Additional file 1. The association between TyG index and thyroid parameters. [file 40001_2023_1501_MOESM1_ESM.doc]

**Figure S1** The association between TyG index and thyroid parameters.


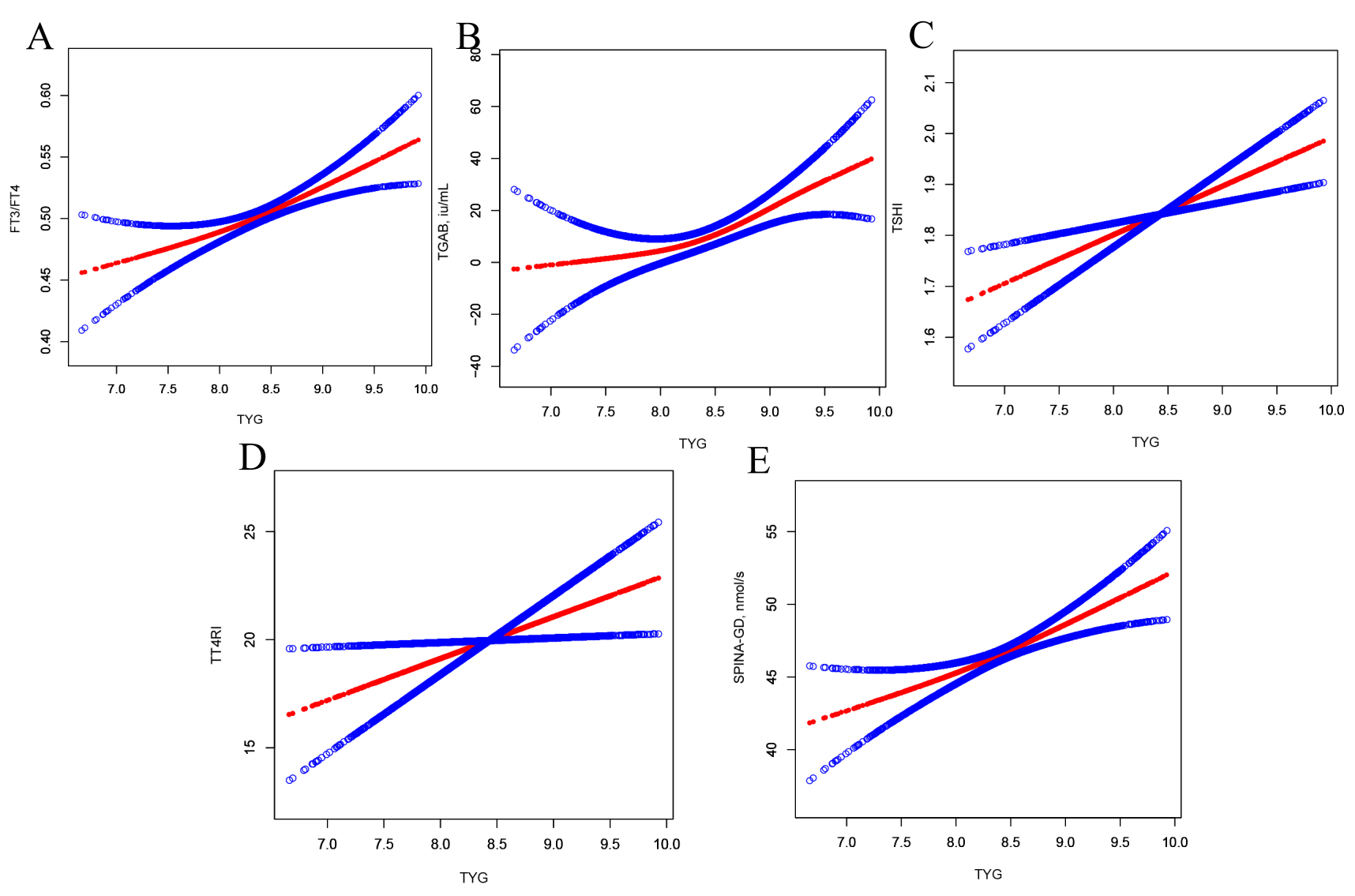


Note: Solid red line represents the smooth curve fit between TyG index and thyroid parameters, including FT3/FT4 (A), TgAb (B), TSHI (C), TT4RI (D), SPINA-GD (E). Blue bands represent the 95% of confidence interval from the fit. Abbreviations: TyG, Triglyceride-glucose; FT3, free triiodothyronine; FT4, free thyroxine; TgAb, anti-thyroglobulin antibody; TSHI, thyrotropin index; TT4RI, thyrotropin thyroxine resistance index. Age, gender, race/ethnicity, education, poverty-to-income ratio, alcohol use, smoke, waist circumference, systolic blood pressure, diastolic blood pressure, physical activity level, hypertension, cardiovascular disease, metabolic syndrome, fasting blood insulin, glycohemoglobin, aspartate aminotransferase, alanine aminotransferase, blood urea nitrogen, high density lipoprotein, low density lipoprotein, total cholesterol, creatinine, uric acid, hemoglobin, and urine iodin were adjusted.
